# Influence of the Shell Thickness and Ratio Between Core Elements on Photostability of the CdTe/CdS Core/Shell Quantum Dots Embedded in a Polymer Matrix

**DOI:** 10.1186/s11671-016-1428-3

**Published:** 2016-04-22

**Authors:** Nataliia Doskaliuk, Yuriy Khalavka, Petro Fochuk

**Affiliations:** Department of Inorganic Chemistry of Solid State and Nanomaterials, Yuriy Fedkovych Chernivtsi National University, Kotsiubynskyi St, 2, Chernivtsi, 58012 Ukraine

**Keywords:** Quantum dots, Core/shell nanocrystals, Photoluminescence, Photooxidation, Layer-by-layer films

## Abstract

This paper reports a study of photooxidation and photomodification processes of the CdTe/CdS quantum dots embedded in a polymer matrix under ambient condition. During the first few minutes of irradiation, the quasi-inverse increase in photoluminescence intensity has been observed indicating the passivation of the nanocrystal surface traps by water molecules. A prolonged irradiation of the polymer film containing CdTe/CdS quantum dots leads to a significant decrease in the photoluminescence intensity together with the “blue shift” of the photoluminescence peak energy associated with quantum dot photooxidation. The mechanisms of the CdTe/CdS core/shell quantum dot photooxidation and photomodification in a polymer matrix are discussed. We have found a correlation between the photostability of the quantum dots and the CdS shell thickness as well as the ratio of core elements.

## Background

Semiconductor nanocrystals or quantum dots (QDs) possess different optical, chemical, and electrical properties from the bulk materials due to quantum confinement effects. QDs are highly luminescent materials with size-dependent emission and absorption spectra [1–3]. They can be used as active materials in optical and optoelectronic devices such as optical switches, sensors, and lasers [4–6]. The light-emitting diodes, lamps, and displays modified with QDs possess a significantly improved color quality [7–9]. A special attention should be paid to solid composites of QDs which are functional materials for luminescence solar light concentrators and spectrum converters [10–13] that are more effective and photostable than those containing organic dyes. The CdTe/CdS QDs are especially interesting in terms of creating luminescence light concentrators because they can be prepared in large quantity in water and have a high fluorescence quantum yield [14]. In addition, CdTe/CdS QDs in aqueous solution have a large effective surface charge that allows their implementation in a polymer matrix with the use of highly flexible and inexpensive layer-by-layer electrostatic assembly [15].

Because of the fact that practical application of QDs composites is mainly related to the interaction with light, the investigation of irradiation influence on the optical properties of QDs in polymer matrix is an important task. Light interaction with QDs can affect the latter in different ways. Very often, high-energy photons launch photoreactions on the surface of such nanocrystals. Under ambient conditions, there are two possible ways of such influence: (i) modification of nanocrystal surface leading to the increase of luminescence intensity and (ii) photooxidation of nanocrystals resulting in degradation of the luminescence spectra. During prolonged irradiation, the combination of both effects is expected.

The oxidation of semiconductors under the above-band irradiation occurs according to the electron-active photooxidation model which suggests the dissociation of molecular oxygen into more active atomic oxygen under the influence of excited charge carriers of QDs [16, 17]. The effect of quantum dot photooxidation has been studied mostly on the CdSe nanocrystals. Those studies indicate the formation of CdSeO_*x*_ (where *x* = 2 or 3) as the photooxidation products [18–20]. Depending on the presence of Cd-bound surface ligand, the physisorbed TeO_2_ layer or CdO and separate TeO_2_ phase can form due to nanocrystals oxidation [21, 22]. The X-ray photoelectron spectroscopy measurement of the CdTe QDs in living cells showed the additional peaks emerging and corresponding to Te–O bonds in CdTeO_3_ after photooxidation [23]. Due to the small difference between the Cd 3d peaks of CdTe and CdO (0.1 eV) with the limitations of the XPS resolution, the detected 0.2 eV shift to lower energy cannot give the clear evidence about the CdO formation. However, the CdO and TeO_2_ were suggested as products of CdTe QDs photooxidation [23].

Despite the fact that the semiconductor nanocrystal oxidation under irradiation is well known, approaches to design photostable QDs have not been thoroughly explored. In this study, we focus on the influence of passivation efficiency of CdTe core by CdS shell and the core element ratio on the photostability of CdTe/CdS QDs embedded in the polymer matrix. The photomodification of the nanocrystals causing initial increase in photoluminescence (Pl) intensity is also discussed briefly.

## Methods

### Synthesis of CdTe/CdS Colloidal Solution

The CdTe QDs colloidal solutions were prepared according to a method proposed by Weller’s group [24]. The method is based on the interaction between cadmium thioglycolate and Te^2−^ anions in an aqueous medium. To prepare the initial precursor, aliquots of 0.01 M 3CdSO_4_·8H_2_O solution and 98 % thioglycolic acid (TGA) from Sigma-Aldrich were mixed and the solution was titrated by 1 M sodium hydroxide until the pH became 11. As a Te^2−^ source, we used electrolytically generated hydrogen telluride which was bubbled through the preceding solution deaerated by argon flow. The final step of the synthesis was CdS shell formation by refluxing the colloid for several hours.

The chemicals used in this study were сadmium sulfate 8/3-hydrate salt, ≥99.0 %; thioglycolic acid ≥98 % (catalog number T3758); sodium hydroxide ≥99 (S8045); and tellurium granular, 99.99 % trace metals basis (263303) all from Sigma-Aldrich.

### Embedding CdTe/CdS QDs in a Polymer Film

Deposition of polymer films containing QDs was carried out on a glass substrate using layer-by-layer assembly proposed by Gero Decher [25]. This technique is based on an electrostatic assembly of oppositely charged materials which are polycation poly(dimethyldiallylammonium chloride) (PDDA) and TGA-stabilized CdTe/CdS QDs in our case. Every QD colloidal solution was diluted to a constant concentration of nanoparticles (2 × 10^−6^ mol/l). The deposition of films was conducted automatically according to the scheme: “immersion of substrate in 0.125 % PDDA aqueous solution—washing in distilled water (1)—immersion in CdTe/CdS colloidal solution—washing in distilled water (2).” One deposition cycle corresponds to the formation of single bilayer “PDDA—QDs” [26]. In this study, the films of 20 bilayers were prepared. We have obtained five series of CdTe/CdS-PDDA multilayer films containing QDs with different CdS shell thickness of three samples in each and three series of polymer films containing QDs with a different core element ratio.

The starting 20 wt% aqueous solution of poly(diallyldimethylammonium chloride) with the average high molecular weight 400.000–500.000 (409030) was from Sigma-Aldrich.

### Film Irradiation and Photoluminescence Measurement

The steady-state photoluminescence measurement was carried out using OceanOptics USB2000 array spectrophotometer. Using SpectaSuite software, we collected Pl spectra of the films and recorded the time dependence of the integrated Pl intensity measured every 10 s. Pl was measured in the course of irradiation of the films by two low pressure mercury lamps with total power of 8 W using optical fiber attached to the side of the films.

## Results and Discussion

**Fig. 1:**
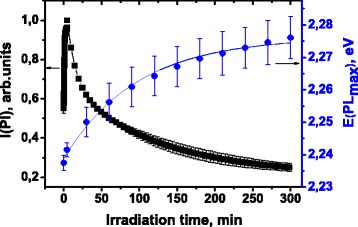
Typical time dependence of the photoluminescence integral intensity (*filled squares*) and the photoluminescence energy maximum (*filled circle*) of the CdTe/CdS quantum dots embedded in polymer matrix during irradiation. The average data obtained from independent measurements of three different samples prepared with the same quantum dots and plotted with *error bars*

Both photomodification and photooxidation processes occur during irradiation of polymer films containing CdTe/CdS QDs with different shell thickness and core element ratio. The photomodification of QDs appears as the significant increase of the Pl integral intensity while the photooxidation is accompanied by the decrease of the Pl intensity and “blue shift” of the Pl energy (Fig. 1).

The first few minutes of irradiation lead to a 50 % increase of the Pl integral intensity and a remarkable “blue shift” of the Pl maximum. There are two proposed explanations of the QDs Pl photoactivation mechanism under short-term irradiation. Cordero et al. suggest that surface adsorbates, specifically water molecules, are responsible for the initial Pl activation of CdSe QDs in monolayer Langmuir film [27]. The irradiation of the QDs monolayer has the effect of quasi-inverse H_2_O molecule physisorption on the nanocrystal surface and passivation of surface traps similar to the enhancement of the luminescence QY observed upon adsorption of electron-donating molecules (Lewis bases) to bulk CdSe surfaces [28]. Wang et al. [29] hypothesized that the initial Pl enhancement of CdSe QDs in densely packed film is mainly due to the Foerster energy transfer process [30]. Because the photooxidation results in a decrease of QDs size and increase of the overlap integral of the absorption and the Pl spectra, the coupling between QDs increases greatly, providing alternative radiative decay channel for the excited carriers.

**Fig. 2:**
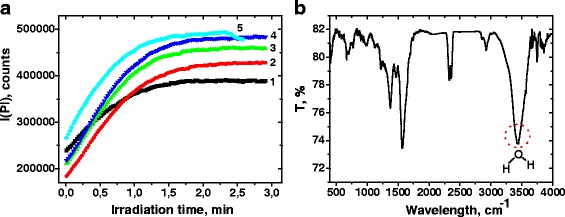
**a** Irradiation time dependence of the CdTe/CdS quantum dot photoluminescence integral intensity by each subsequent cycle of photoactivation from *1* to *5*. After reaching the maximum of photoluminescence intensity, the irradiation source was turned off. Each following curve was recorded after 30 min of storage of the sample without irradiation. **b** FTIR spectrum of PDDA-CdTe/CdS multilayer film

We assume that the main difference between those two mechanisms which can be figured out experimentally is the reversibility of the photomodification process. If the initial increase of the Pl intensity is associated with the coupling strengthening due to QDs oxidation, the photoactivated state should be irreversible. In contrast, reversible desorption of water molecules during irradiation should lead to the recovery of the initial value of intensity. In order to verify the reversibility of the initial Pl activation, we blocked the light from irradiation source when Pl intensity reached its maximum and measured Pl again, after storage of the sample in the dark for 30 min. The increase of the Pl integral intensity under short-term exposure was quasi-inverse in the case of CdTe/CdS quantum dots in polymer matrix (Fig. 2a).

This observation supports the conclusion about passivation of surface traps by physisorbed H_2_O molecules. The presence of a large amount of H_2_O in PDDA-CdTe/CdS films was confirmed by the observation of the band of asymmetric stretching of OH group in the Fourier transform infrared spectroscopy (FTIR) spectra at 3440 sm^−1^ (Fig. 2b). With each subsequent cycle of short-term exposure, the Pl integral intensity increases indicating the formation of extra surface traps. In addition, the increase of the Pl energy maximum has been observed during photoactivation (Fig. 1). Therefore, we propose that the fast oxidation of the most active surface center accompany the surface modification by H_2_O molecules. In our opinion, the most suitable candidates for such centers are the anion vacancies or the Cd dangling bonds. The anion vacancies oxidation is the most thermodynamically favorable process in the system and can be described by the scheme:

1 $$ \mathrm{C}\mathrm{d}\mathrm{T}{\mathrm{e}}_{1\hbox{-} \mathrm{n}}{\mathrm{V}}_{\mathrm{Te}\ \mathrm{n}}/\mathrm{C}\mathrm{d}\mathrm{S} + \left[\mathrm{O}\right]\ \to\ \mathrm{C}\mathrm{d}\mathrm{T}{\mathrm{e}}_{1\hbox{-} \mathrm{n}}{\mathrm{O}}_{\mathrm{n}}/\mathrm{C}\mathrm{d}\mathrm{S} $$

The CdTeO_*x*_ formation can create extra non-radiative levels in the QDs band structure resulting in the quasi-inverse Pl intensity changes during brief irradiation. The QDs surface modification by H_2_O molecules and the photooxidation of nanocrystals are competing processes during the long-term irradiation, but the second one begins to dominate when the critical concentration of chemisorbed oxygen atoms is reached.

**Fig. 3:**
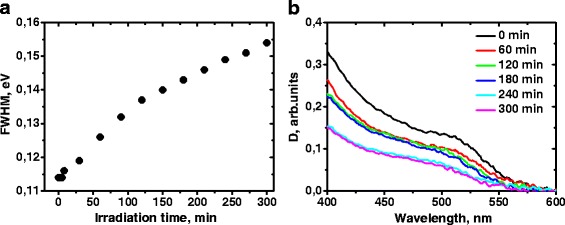
**a** Dependence of full width at half maximum of the photoluminescence spectra of CdTe/CdS quantum dots embedded in polymer matrix on the irradiation time. **b** Absorption spectra of CdTe/CdS quantum dots in polymer matrix recorded after different irradiation time

Further irradiation of the sample for 5 h leads to the Pl spectra degradation together with increase of the Pl energy maximum (Fig. 1). In addition, the increase in the full width at a half maximum and the degradation of the absorption spectrum have been observed indicating the photooxidation of the nanocrystals (Fig. 3).

In order to prevent such undesirable phenomena, enhancement of the passivation efficiency of the CdTe core was proposed. As shown above, the photooxidation rate is much slower in the case of the CdSe/ZnS core/shell NCs compared to the CdSe nanocrystals under environments containing O_2_ [31]. The significantly longer time scale stability was observed in the case of CdSe/ZnS QDs with seven ZnS layers compared with those with five layers [32]. Given these results, it can be assumed that the shell thickness is an important factor that may affect the photostability of core/shell nanocrystals.

**Fig. 4:**
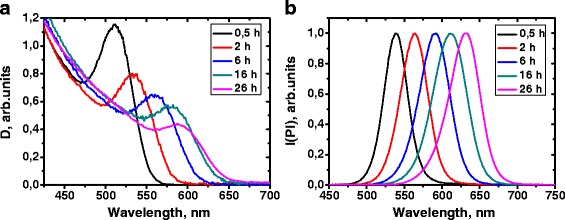
Absorption (**a**) and normalized photoluminescence spectra (**b**) of the CdTe/CdS quantum dot colloidal solution with different thermal treatment times

In order to investigate the influence of the CdS shell thickness on the CdTe/CdS QDs photostability, the starting colloidal solution of TGA-stabilized CdTe QDs with Cd/Te molar ratio of 1:0.3 was refluxed during 26 h with the sampling of aliquots after 0, 5, 2, 6, and 16 h. It is known that the refluxing of thiol-stabilized CdTe QDs colloidal solutions leads to cadmium-thiolates molecule hydrolysis resulting in release of S^2−^ anions and forming of CdS shell on the Cd-reach surface of nanocrystals [33]. Therefore, the shell thickness increases during the thermal treatment. The growth of CdS shell can be monitored by optical spectroscopy. With increasing reflux time, the absorption and the Pl maximum shifts towards longer wavelength indicating an increase of the QDs diameter due to the CdS shell growth (Fig. 4).

**Fig. 5:**
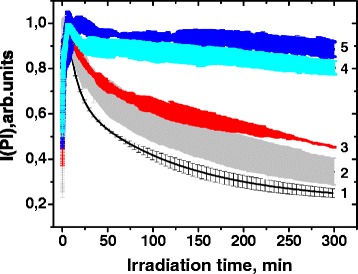
Irradiation time dependence of the photoluminescence integral intensity of the CdTe/CdS quantum dots with different passivation times: 1–0.5, 2–2, 3–6, 4–16, and 5–26 h. Lines are averaged through measurement of three different samples prepared with the same quantum dots and are presented with *error bars*

The irradiation of CdTe/CdS-PDDA multilayer films containing QDs with different CdS shell thickness has demonstrated clear correlation between their photostability and refluxing (core passivation) time (Fig. 5).

The rate of the Pl intensity drop decreases significantly with the increase of QDs passivation time. The first three kinetic curves corresponding to the thermal treatment period up to 6 h can be fit well by the biexponential decay function with the average time constants 155, 230, and 255 min, respectively. Further increase of the passivation time leads to a slight and almost linear decrease of the Pl intensity under irradiation. These facts indicate effective protection of CdTe core by CdS shell from photooxidation. We can also assume that only the CdTe core is mainly involved in the photooxidation of CdTe/CdS QDs. This assumption is based on the calculation of Gibbs energy change for CdTe (∆*G* = −406.7 kJ/mol) and CdS (∆*G* = −372.1 kJ/mol) oxidation reactions—the oxidation of CdTe is more thermodynamically favorable. On the other side, the excited electrons in CdTe/CdS QDs are delocalized throughout all heterostructure due to a similar value of lower occupied molecular orbital of the core and shell which may promote the shell oxidation. The long refluxing and increase in the shell thickness leads to the type II heterostructure formation when the excited electrons are localized in the shell [33]. The probability of its photooxidation should increase in this case. In contrast, we observed the significant increase in the CdTe/CdS QDs photostability with increase in CdS shell thickness indicating the thermodynamic limitation of the photooxidation process.

**Fig. 6:**
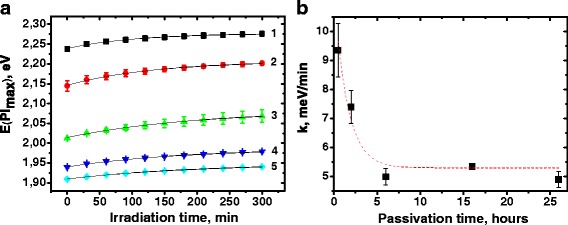
**a** Irradiation time dependence of photoluminescence energy maximum of the polymer composites containing CdTe/CdS quantum dots with different passivation times: 1–0.5, 2–2, 3–6, 4–16, and 5–26 h. **b** Dependence of the Pl energy maximum shift rate on the passivation time of quantum dots

The change of the Pl energy maximum under irradiation is also non-linear in all cases (Fig. 6a).

The best coefficient of determination was obtained by the fitting of the experimental data with monomolecular exponential growth function:

$$ y=A\left(1-{e}^{-k\left(x-xc\right)}\right), $$

where *k* is the rate of the Pl energy maximum shift. The rate of the Pl energy maximum shift decreases two times with the increase in the passivation time for 6 h and then remains almost unchanged (Fig. 6b).

**Fig. 7:**
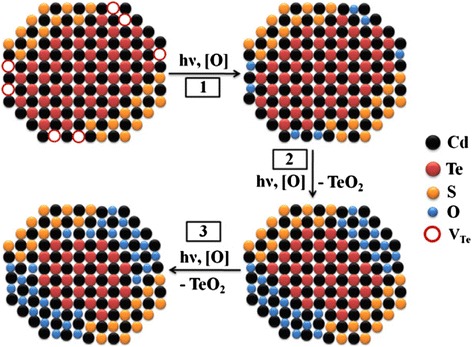
Schematic representation of the three-step photooxidation process of CdTe/CdS core/shell nanocrystal: *1*—the anion vacancies oxidation, *2*—oxidation of bare CdTe core, and *3*—oxidation of passivated area of the core due to oxygen diffusion through the CdS shell

It was suggested that the core photooxidation may be possible due to oxygen diffusion through the shell in core/shell nanocrystals [32, 34]. Due to large lattice mismatch of zinc-blende CdTe and CdS (11.3 %), the formation of defects such as low-angle boundaries or grain boundaries is possible that significantly accelerates the oxygen diffusion to the core. The photooxidation products may even rupture the shell resulting in the rapid reduction of the QDs size. We propose that at the short refluxing times, incomplete passivation of the core occurs similarly to the oxidation observed for the CdSe core partially coated with ZnS [18]. According to our results, a complete CdS shell is formed in 6 h through refluxing. A slight photooxidation of the complete passivated nanocrystals is related to the oxygen diffusion through the shell. The combination of these two processes may predetermine the biexponential Pl decay and nonlinear change of the FWHM and the Pl energy maximum of the QDs with refluxing time of up to 6 h. The first time constant is one order less than the second in all cases. Therefore, we propose that it is related to fast photooxidation of uncovered areas of the core which are more able to the react with oxygen. At the same time, the formation of the CdO on top of those areas may prevent further photooxidation of QDs. The large second time constant can be attributed to the oxidation of the passivated area of the core due to the slow oxygen diffusion through the shell. It should be emphasized that the fast oxidation of the anion vacancies is preceded or precedes (with no “by” following it) by these two stages. Hence, we can propose the three-step photooxidation model of CdTe/CdS core/shell QDs embedded in polymer matrix (Fig. 7).

During the first few minutes of irradiation, the fast oxidation of anion vacancies accompanied by the H_2_O molecules physisorption occurs. At the second stage, the bare CdTe core is oxidized resulting in a fast Pl decay and sufficient decrease of core radii due to TeO_2_ formation. The third photooxidation stage caused by oxygen diffusion through the shell results in a slow Pl decay and QDs radii decrease.

Taking into account that the uncovered area of CdTe core is highly reactive in the photooxidation process but optical properties of such nanocrystals are desirable in some cases, we propose to reduce the core reactivity by decreasing its defectiveness. By changing the stoichiometric composition of CdTe clusters, we can obtain the nanocrystals with different Te anion vacation concentration in the core. When the other synthesis conditions including Cd/TGA ratio, refluxing time, and pH remain constant, the passivation degree of this vacancies increases approaching the stoichiometric Cd/Te ratio. The short-term refluxing, insufficient to complete passivation, is necessary to obtain the different anion vacancy concentrations in the prepared CdTe/CdS QDs.

**Fig. 8:**
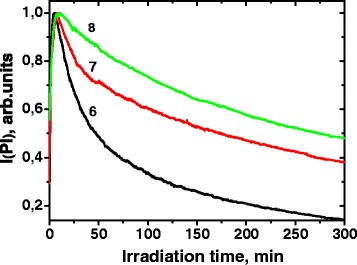
Irradiation time dependence of photoluminescence integral intensity of CdTe/CdS quantum dots with different Cd/Te ratios: 6:20, 7:4, 8:2. The refluxing time of the crude colloidal solution was 2 h

Photoinduced Pl decay curves of the CdTe/CdS QDs with different Cd/Te ratio embedded in polymer films are shown in Fig. 8.

Just like in the previous experiment, the dependence of Pl intensity on irradiation duration can be fit well by biexponential decay function with the average time constant 155 (curve 6), 230 (curve 7), and 255 min (curve 8). Therefore, the decrease in the Cd/Te ratio and approaching to the stoichiometric core composition results in a remarkable decrease of the photooxidation rate. This observation confirms that the anion vacancies are involved in the photooxidation of the CdTe/CdS QDs in accordance with scheme 1. As has been discussed above, the vacancy oxidation occurs very fast during the first minutes of irradiation, and therefore, it does not appear on the part of kinetic curves relating to the photooxidation. Probably, the dependence of the photooxidation rate on the vacancies concentration is caused by formation of CdTe_1−n_O_n_ fragments which acts as active centers of further Te^2−^ anions oxidation due to the shift of the electron density to oxygen and weakening of the Cd-Te bonds.

### Conclusions

The photooxidation and photomodification of the CdTe/CdS quantum dots embedded in the polymer matrix has been investigated. The quasi-inverse photoluminescence intensity increase has been observed during the first few minutes of UV irradiation indicating the surface trap passivation by H_2_O molecules accompanied by the anion vacancy oxidation. Further irradiation has led to quantum dot photooxidation manifested as a significant “blue shift” of the Pl energy maximum and the decrease in photoluminescence intensity. The increase in the CdS shell thickness and decrease in Cd/Te ratio lead to a significant increase in the CdTe/CdS quantum dot photostability. The three-step mechanism of CdTe/CdS quantum dot photooxidation including anion vacancies oxidation, oxidation of the bare core area, and slow core oxidation via oxygen diffusion through the shell has been proposed.

## Abbreviations

FTIR: Fourier transform infrared spectroscopy
FWHM: full width at half maximum
PDDA: poly(dimethyldiallylammonium chloride)
Pl: photoluminescence
QDs: quantum dots
TGA: thioglycolic acid
